# Supplementary material for: Content-rich biological network constructed by mining PubMed abstracts
Source: BMC Bioinformatics. 2004 Oct 8;5:147. doi: 10.1186/1471-2105-5-147 (PMC528731; doi:10.1186/1471-2105-5-147)
Supplement: Additional File 2 — The original results of the above study (non-essential files are deleted to keep the file size under the limit set by BMC bioinformatics). [file 1471-2105-5-147-S2.bz2 › chilibotAdditionalFile2/dip50/572ID10722747E10939/html/NTRK1_BDNF.html]

 


 **NTRK1** and **BDNF** 
  
Found 566 abstracts in PubMed, retrieved 50.  
 

 What does Google say? 
 PDF only 
| .edu only 

---

**Interactive relationship** (e.g. stimulation, inhibition, etc)

**Inhibitory relationship**- The suppressive effect of  **BDNF**  on LTD was prevented by either the tyrosine kinase Trk  [ **NTRK1** ]  receptor inhibitor K252a or the phospholipase C inhibitor U73122.  Ref: 12153539 Eur J Neurosci, 2002
- Moreover, the neuroprotective effect of  **BDNF**  and LIGA20 was abolished in neurons exposed to the Trk  [ **NTRK1** ]  tyrosine kinase inhibitor k252a, demonstrating a relationship between neuroprotection and activation of Trk  [ **NTRK1** ]  receptors.  Ref: 12829403 Neurotox Res, 2002
**Neutral relationship**- In Shc signaling, NGF, but not  **BDNF** , displayed interactions between Trk  [ **NTRK1** ]  and Shc in a phenylarsine oxide dependent manner in TrkB expressing PC12 cells.  Ref: 12419535 Brain Res, 2002
- Here we show that p62 interacts not only with TrkA  [ **NTRK1** ] , but also with TrkB and TrkC, which are the primary receptors for brain derived neurotrophic factor  [ **BDNF** ]  and neurotrophin 3.  Ref: 12471037 J Biol Chem< MedlineTA>J Biol Chem, 2002
- Nerve growth factor, brain derived neurotrophic factor  [ **BDNF** ]  and neurotrophin 3, members of the neurotrophin family, bind to and activate TrkA  [ **NTRK1** ] , TrkB and TrkC, respectively, members of the Trk  [ **NTRK1** ]  receptor tyrosine kinase family, to exert various effects including promotion of differentiation and survival, and regulation of synaptic plasticity in neuronal cells.  Ref: 12419535 Brain Res, 2002
- Trk  [ **NTRK1** ]  receptors are a family of three receptor tyrosine kinases, each of which can be activated by one or more of four neurotrophins nerve growth factor, brain derived neurotrophic factor  [ **BDNF** ] , and neurotrophins 3 and 4.  Ref: 12676795 Annu Rev Biochem< MedlineTA>Annu Rev Biochem, 2002
- To gain insight into the effects of DR on expression of nerve growth factor and the functional state of NT signalling pathways, we assessed the phosphorylation state of Trk  [ **NTRK1** ]  receptors in light reared animals, in dark reared animals, in DR animals briefly exposed to light and in DR animals with exogenous supply of NTs NGF, brain derived neurotrophic factor  [ **BDNF** ]  and NT 4 in the VC.  Ref: 12453056 Eur J Neurosci, 2002
- **BDNF**  stimulated much stronger interactions of PLCgamma with Trk  [ **NTRK1** ]  than NGF and NT 3 in PC12 cells stably expressing TrkB and cultured cerebral cortical neurons, respectively, although  **BDNF** , NGF and NT 3 induced similar levels of tyrosine phosphorylation of Trk  [ **NTRK1** ] .  Ref: 12419535 Brain Res, 2002

**Non-interactive relationship** (e.g. studied together, co-existance, homology, etc.)

- To test this, we infused the cortex with an inhibitor of Trk  [ **NTRK1** ]  receptor tyrosine kinases, anti TrkB IgG1, anti  **BDNF** , and anti neurotrophin 4 5 antibodies.  Ref: 12736347 J Neurosci, 2003
- Both flat and process bearing ECs were immunopositive for brain derived neurotrophic factor  [ **BDNF** ] , nerve growth factor, neurotrophin 4, and TrkB but not TrkA  [ **NTRK1** ] .  Ref: 12555206 Glia, 2003
- Brain derived neurotrophic factor  [ **BDNF** ]   **BDNF**  and its receptor, tyrosine receptor kinase trk  [ **NTRK1** ]  B, play important roles in neural plasticity, long term potentiation and memory formation.  Ref: 12473084 Eur J Neurosci, 2002
- We found that 1 trk  [ **NTRK1** ]  C, a receptor for neurotrophin 3 NT 3, is dominantly expressed in RCEC, but trk  [ **NTRK1** ]  B, a receptor for brain derived neurotrophic factor  [ **BDNF** ] , is dominantly expressed in RAEC.  Ref: 12745089 Biochem Biophys Res Commun, 2003
- In the present study, we examined human follicular fluid and mouse immature oocytes to determine whether another Trk  [ **NTRK1** ]  B ligand, neurotrophin 4 5 NT 4 5, is present within the ovarian follicle and if so, whether it demonstrates activity similar to that of  **BDNF** .  Ref: 12364436 J Clin Endocrinol Metab, 2002
- While  **BDNF** , likely acting through the Trk  [ **NTRK1** ]  B receptor, was necessary for the induction of long lasting Ca2 activated K currents via intracellular Ca2 elevation,  **BDNF**  was not necessary for the maintenance of this current.  Ref: 12405954 Eur J Neurosci, 2002
- **BDNF**  and Trk  [ **NTRK1** ]  B  **BDNF**  receptor mRNAs were determined by RNAse protection assay.  Ref: 12470709 Neurochem Int, 2003
- The ability of  **BDNF**  to rescue the cells from chemotherapeutic agent induced cell death was inhibited by treatment with the Trk  [ **NTRK1** ]  tyrosine kinase inhibitor K252a or the phosphatidylinositol 3 kinase inhibitor LY294002, but not by the mitogen activated protein kinase kinase inhibitor PD98059 or the peritoneal lymphocyte gamma inhibitor U73122, indicating that both TrkB and PI3K activities are required for the survival promoting effects of  **BDNF** .  Ref: 12438277 Cancer Res, 2002
- To determine which of the neurotrophins NTs nerve growth factor NGF, brain derived neurotrophic factor  [ **BDNF** ]   **BDNF** , neurotrophin 3 NT3, and neurotrophin 4 5 NT4 and their receptors NTrs, TrkA  [ **NTRK1** ] , TrkB, TrkC, and p75, are present in the adult rat lacrimal gland.  Ref: 12882781 Invest Ophthalmol Vis Sci, 2003
- New findings were RT PCR for neurotrophins and their receptors detected NT 3 and NT 4 5 in alveolar macrophages,  **BDNF** , NT 4 5, trkA  [ **NTRK1** ] , the truncated form of trkB, and trkC in lung homogenate, but no trks in alveolar macrophages, immunohistochemistry for neurotrophin receptors detected TrkA  [ **NTRK1** ]  in capillary cells, the truncated form of TrkB, and TrkC in interstitial macrophages, immunoelectron microscopy for TrkC revealed expression of TrkC on the surface of interstitial macrophages, and in situ hybridization for neurotrophins detected  **BDNF**  in interstitial macrophages and alveolar type I cells, NT 3 in alveolar macrophages, and NT 4 5 in alveolar and interstitial macrophages.  Ref: 12122447 Histochem Cell Biol, 2002
- Lithium neuroprotection against excitotoxicity is inhibited by a  **BDNF**  neutralizing antibody and K252a, a Trk  [ **NTRK1** ]  antagonist.  Ref: 12701214 Seishin Shinkeigaku Zasshi, 2003
- Longitudinal sections of grafts were immunostained by means of the following antibodies anti NGF, anti  **BDNF** , anti trkA  [ **NTRK1** ] , and anti p75.  Ref: 12404348 Microsurgery, 2002
- We used a semiquantitative reverse transcription polymerase chain reaction technique to measure the messenger RNA for neurotrophins  **BDNF**  and NGF, and for their receptors trkB, trkA  [ **NTRK1** ] , and the low affinity receptor, p75 in the hippocampus and basal forebrain of rats after 28 weeks of alcohol consumption without malnutrition.  Ref: 12231238 Brain Res, 2002
- NGF infusion results in elevated levels of  **BDNF**  expression in both intact and injured trkA  [ **NTRK1** ]  positive neurons, accompanied by reduced trkB expression.  Ref: 12405958 Eur J Neurosci, 2002
- Moreover, trophic effects of OLG conditioned medium were partially blocked by K252a, a trk  [ **NTRK1** ]  tyrosine kinase inhibitor, and by neutralizing anti  **BDNF**  or anti NT 3 antisera, indicating that neurotrophins may mediate these effects, perhaps in concert with other signals.  Ref: 12843289 J Neurosci, 2003
- The  **BDNF**  receptor, Trk  [ **NTRK1** ]  B, was identified in mouse oocytes, and  **BDNF**  accelerated first polar body extrusion in vitro.  Ref: 12364436 J Clin Endocrinol Metab, 2002
- OBJECTIVE There are preclinical data showing that fluoxetine stimulated expression of Brain Derived Neurotrophic Factor  [ **BDNF** ]   **BDNF**  and its specific tyrosine kinase receptor  [ **NTRK1** ] , and caused neuritic elongation and increased dendritic branching density of CA3 hippocampal pyramidal cell neurons in rodents.  Ref: 12779183 Int J Psychiatry Med, 2002
- K252a, an inhibitor of Trk  [ **NTRK1** ]  tyrosine kinases, and a  **BDNF**  neutralizing antibody suppressed the neuroprotective effect of lithium.  Ref: 12504924 Neuropharmacology, 2002
- We investigated whether changes in  **BDNF**  and trk  [ **NTRK1** ]  B mRNA expression occurred in both olfactory and visual processing systems at 4.5 h postpartum, 2 3 h after the behavioural manifestations of an olfactory recognition memory were found.  Ref: 12473084 Eur J Neurosci, 2002
- Increased  **BDNF**  and trk  [ **NTRK1** ]  B mRNA expression in cortical and limbic regions following formation of a social recognition memory.  Ref: 12473084 Eur J Neurosci, 2002
- Low levels of brain derived neurotrophic factor  [ **BDNF** ]  and tyrosine kinase receptor  [ **NTRK1** ]  B are related to loss of dentate granule cells after prolonged low protein feeding in the rat.  Ref: 12231435 Neurosci Lett, 2002
- Cultured cortical progenitors express  **BDNF**  and NT 3, as well as their receptors TrkB tyrosine kinase receptor  [ **NTRK1** ]  B and TrkC.  Ref: 12832539 J Neurosci, 2003
- By 1 week and up to 3 weeks later expression is still seen in approximately 66% of injured neurons, but the characteristic phenotypic switch in the subpopulations expressing  **BDNF**  occurs, whereby expression in the trkA  [ **NTRK1** ]  population is reduced and expression in trkB and in trkC positive neurons is elevated.  Ref: 12405958 Eur J Neurosci, 2002
- Expression of the tyrosine kinase receptor  [ **NTRK1** ]  that selectively binds  **BDNF** , trkB, is increased in the spinal dorsal horn during inflammation as well.  Ref: 12435470 Pain, 2002
- Furthermore, when the TrkB tyrosine kinase was blocked with the Trk  [ **NTRK1** ]  specific inhibitor CEP 2563, or by neutralizing antibody to  **BDNF** , sensitivity to chemotherapy was significantly increased.  Ref: 12438236 Cancer Res, 2002
- The aim of this study was to verify the expression of two neurotrophins, nerve growth factor and brain derived neurotrophic factor  [ **BDNF** ] , as well as their receptors, trkA  [ **NTRK1** ]  and p75, in an early stage of axonal regeneration in muscle vein combined grafts.  Ref: 12404348 Microsurgery, 2002
- We suggested that these effects could result from alterations in the levels of brain derived neurotrophic factor  [ **BDNF** ]   **BDNF**  and tyrosine kinase receptor  [ **NTRK1** ]  B TrkB.  Ref: 12231435 Neurosci Lett, 2002
- In this work the expression and anatomical localization of NGF,  **BDNF**  and NT 3 and of corresponding high affinity receptors TrkA  [ **NTRK1** ] , TrkB full length and truncated TR isoforms, TrkC and of the low affinity p75 receptor were assessed in surgical samples from adult human lung by reverse transcriptase polymerase chain reaction RT PCR, Western blot and immunohistochemistry.  Ref: 12791675 Am J Respir Cell Mol Biol< MedlineTA>Am J Respir Cell Mol Biol, 2002
- **BDNF**  s ability to rescue the cells from cisplatin induced cell death was inhibited by treatment with the Trk  [ **NTRK1** ]  tyrosine kinase inhibitor, K252a, and the phosphatidylinositol 3 kinase 3 kinase inhibitor, LY294002.  Ref: 12691830 Cancer Lett, 2003
- Western blot analysis confirmed these results, showing that the lacrimal gland expresses NGF,  **BDNF** , NT3, and NT4 as well as the NTrs TrkA  [ **NTRK1** ] , TrkB, and TrkC and the p75 protein.  Ref: 12882781 Invest Ophthalmol Vis Sci, 2003
- In contrast, null mutant mice for TrkB, the tyrosine kinase receptor  [ **NTRK1** ]  for brain derived neurotrophic factor  [ **BDNF** ]  and neurotrophin 4, displayed a clear loss of locus coeruleus noradrenergic neurons.  Ref: 12810600 Development, 2003
- RT PCR showed positive bands at the appropriate sizes for NGF,  **BDNF** , NT3, and NT4, and for the receptors TrkA  [ **NTRK1** ] , TrkB, TrkC, and p75.  Ref: 12882781 Invest Ophthalmol Vis Sci, 2003
- These effects suggest NGF can regulate  **BDNF**  expression in trkA  [ **NTRK1** ]  expressing neurons regardless of the axonal state.  Ref: 12405958 Eur J Neurosci, 2002
- These findings demonstrate that by 2 3 h following the initial formation of olfactory recognition memory there are  **BDNF**  trk  [ **NTRK1** ]  B mediated plasticity changes in brain areas involved in the consolidation of olfactory memory the pyriform and entorhinal cortices.  Ref: 12473084 Eur J Neurosci, 2002
- Many of these cells reacted for NGF,  **BDNF** , and p75, but not trkA  [ **NTRK1** ] .  Ref: 12404348 Microsurgery, 2002
- Consistent with the presence of trkB but not trkA  [ **NTRK1** ] ,  **BDNF**  causes rapid phosphorylation of MAP kinases ERK1 and ERK2, but NGF does not.  Ref: 12672017 J Neurobiol, 2003
- GABA decreased the glutamate induced augmentation with no effect on the expression of mRNA encoding the  **BDNF**  receptor, Trk  [ **NTRK1** ]  B.Measurements of  **BDNF**  transcripts levels showed that transcripts containing exons I and III were increased by picrotoxin.  Ref: 12470709 Neurochem Int, 2003
- As the signal transducing receptor for  **BDNF** , the full length 145 tyrosine kinase receptor  [ **NTRK1** ]  trk  [ **NTRK1** ]  B, is expressed in multiple sclerosis lesions, it is likely that the  **BDNF**  secreted by GA reactive TH2 and TH1 has neurotrophic effects in the multiple sclerosis target tissue.  Ref: 12390966 Brain, 2002
